# Endo-Periodontal Lesions—An Overlooked Etiology of Odontogenic Sinusitis

**DOI:** 10.3390/jcm12216888

**Published:** 2023-10-31

**Authors:** Jianyou Wu, Ming Zheng, Xiangdong Wang, Songlin Wang

**Affiliations:** 1 Beijing Stomatological Hospital, Capital Medical University, Tian Tan Xi Li 4#, Beijing 100050, China; dentistwu@126.com; 2 Beijing Laboratory of Oral Health, Capital Medical University, Tian Tan Xi Li 4#, Beijing 100050, China; 3 Department of Stomatology, Beijing TongRen Hospital, Capital Medical University, Dongjiaominxiang, Beijing 100730, China; 4 Department of Otolaryngology–Head and Neck Surgery, Beijing TongRen Hospital, Capital Medical University, Dongjiaominxiang, Beijing 100730, China; entzheng@aliyun.com; 5 Beijing Key Laboratory of Nasal Diseases, Beijing Institute of Otorhinolaryngology, Dongjiaominxiang, Beijing 100730, China

**Keywords:** odontogenic sinusitis, oral etiology, endo-periodontal lesions, clinical symptoms

## Abstract

The aim of this study was to analyze the oral etiology of patients with odontogenic sinusitis (ODS) and to compare the differences in demographic data, clinical symptoms, extent of sinus involvement, bone penetration of the maxillary sinus floor (MSF) between different etiologies. A retrospective investigation was conducted on 103 patients with ODS recruited from Beijing TongRen Hospital. All enrolled patients underwent sinus CT, nasal endoscopy, and oral examination. A comparison of the patients’ clinical symptoms, the extent of involvement of the sinuses, and bone resorption of the MSF according to odontogenic etiologies was conducted. Follow-up was based on symptoms and clinical examination. The most common odontogenic etiologies were endo-periodontal lesions (EPLs, 49.5%), apical periodontitis (AP, 32.0%), and periodontitis (PE, 8.7%). There were statistically significant differences in age (*p* = 0.002), sex (*p* = 0.036), inflammation involving the ethmoid sinus (*p* = 0.037), and bone penetration of the MSF (*p* < 0.001) between the AP, EPL, and PE groups. There were no significant differences in sinusitis symptoms (*p* > 0.005) among patients with different odontogenic etiologies. In conclusion, EPL is a neglected oral etiology with a destructive effect on the bone of the MSF, which deserves more attention in diagnosis and treatment.

## 1. Introduction

Odontogenic sinusitis (ODS) is a bacterial sinusitis, an infection of dental origin that starts in the lower wall of the maxillary sinus and can be caused by periodontitis (PE), apical periodontitis (AP), oral maxillary sinus fistula and maxillary cysts, or secondary to complications of dental treatment [1]. In recent years, the number of papers on ODS has increased, although there is a lack of common diagnostic names and criteria. Most of them use “odontogenic sinusitis”, some use “odontogenic maxillary sinusitis”, and a few use the newer classification “sinonasal complications of dental disease and treatment” [2]. It is now clear that ODS is distinct from non-odontogenic sinusitis, which is classified as unilateral secondary chronic sinusitis due to localized odontogenic lesions, according to the European Position Paper on Rhinosinusitis and Nasal Polyps 2020 (EPOS) [3]. Misdiagnosis of the cause of sinusitis could lead to incorrect treatment, and if the sinus and odontogenic pathological irritation are not treated simultaneously, it may lead to treatment failure [4]. Currently, there are no clear definitions and ideal treatments for ODS. Some scholars believe that diagnosis of ODS does not require symptoms of sinusitis, emphasizing that nasal endoscopy is more effective in confirming bacterial sinusitis [5]. However, given that both the EPOS and American Academy of Otolaryngology–Head and Neck Surgery guidelines require specific symptoms for the diagnosis of chronic sinusitis, the adjunctive support of endoscopy and computed tomography (CT) imaging may not be fully representative of the ODS population [6]. In addition, in the early stage of ODS, oral lesions cause inflammation of the sinus mucosa, and in the absence of obstruction of the maxillary sinus orifice, the majority of patients do not have obvious symptoms of sinusitis and are found to have thickening of the mucosa of the maxillary sinus upon cone-beam computed tomography (CBCT) examination, which should be called odontogenic maxillary sinusitis to be more precise and has been reported extensively by oral surgeons and radiologists [7,8,9,10,11,12]. The severity of mucosal thickening is directly proportional to the oral lesion, and by treating the oral lesion, the sinus mucosal thickening can be controlled or even normalized. In our study, all patients underwent multidisciplinary evaluation, where otorhinolaryngologists specified the clinical signs of sinusitis and completed nasal endoscopy and sinus CT and dentists performed oral examinations and diagnosis of oral lesions. Previous studies have compared sinus symptoms and imaging findings in ODS with those in non-ODS, and they found that foul smell, ipsilateral facial pressure, and middle meatal pus on endoscopy correlated higher with ODS than non-ODS [13,14]. However, the effects of different odontogenic etiologies on the symptoms of sinusitis have rarely been reported. Therefore, this study aims to analyze the effects of different odontogenic etiologies on the clinical symptoms, the extent of sinus involvement, and bone resorption and penetration in the maxillary sinus floor (MSF) among patients with ODS.

## 2. Materials and Methods

### 2.1. Clinical Data and Sample Collection

A total of 103 patients with ODS recruited from the Department of Otolaryngology and Stomatology of Beijing TongRen Hospital between 2019 and 2021 were included in this study. All patients underwent endoscopic sinus surgery (ESS), and 76 of 103 patients completed ESS and stomatological surgery simultaneously (including oral lesion tooth extraction, as well as implant removal and oral maxillary sinus fistula repair). Sinus CT, nasal endoscopy, and oral examination were conducted for all the patients, and CBCT was performed for some patients with odontogenic lesions that could not be assessed on sinus CT images. The oral examinations included assessment of tooth condition or restoration, endodontic electrical vitality measurement, periodontal pocket depth (PPD), clinical attachment loss (CAL), and bone resorption of the MSF (based on CT images). The regular ENT examination included the patient’s pre-operation and post-operation sinusitis symptoms and nasal endoscopy, which was evaluated on the basis of the modified Lund–Kennedy endoscopic scoring system, retaining the subscores of polyps, edema, and discharge but excluding the subscores of scarring and crusting [15].

### 2.2. Inclusion and Exclusion Criteria

Clinical symptoms of chronic rhinosinusitis (obstruction, nasal discharge, facial pain/pressure, and reduction or loss of smell) and imaging findings (CT scans showing hypodense occupancy in the maxillary sinus and bony discontinuity of the MSF) were the inclusion criteria. In addition, the included patients also had an oral lesion corresponding to the maxillary sinus lesion.

Patients meeting one of the following criteria were excluded from the study: patients with maxillary sinus lesions without symptoms of sinusitis, those with only partial mucosal thickening of the maxillary sinus, those with fungal bulb sinusitis on imaging, and those with benign or malignant tumors of the maxillary sinus.

### 2.3. Diagnostic Criteria for Odontogenic Lesions

The following findings characterize the diagnostic criteria for odontogenic lesions:

1. Endo-periodontal lesions (EPLs): vitality of dental pulp (−); CAL (+); PPD (>3 mm); and radiological bone loss (+) [16,17].
2. Apical periodontitis (AP): vitality of dental pulp (−); CAL (−); PPD (≤3 mm); and radiological bone loss (−) [18].
3. Periodontitis (PE): vitality of dental pulp (+); interdental CAL detectable at ≥2 nonadjacent teeth; buccal or oral CAL ≥ 3 mm with pocketing ≥ 3 mm detectable in ≥2 teeth, although the observed CAL cannot be ascribed to non-PE-related causes, such as (1) gingival recession of traumatic origin, (2) dental caries extending in the cervical area of the tooth, (3) CAL on the distal aspect of a second molar associated with malposition or extraction of a third molar, (4) endodontic lesion draining through the marginal periodontium, and (5) vertical root fracture [17].
4. Temporary oroantral communications: oral maxillary sinus communication after tooth extraction, mucosal healing of the oral fistula, and bone loss at the base of the maxillary sinus [19].
5. Permanent oroantral fistulas: oral maxillary sinus communication after tooth extraction, unhealed intraoral fistula, and oral and maxillary sinus communication [19].

### 2.4. Surgical Procedures

All enrolled ODS patients underwent endoscopic sinus surgery (ESS) under general anesthesia. The routine surgical procedure included uncinectomy, identification, and enlargement of the maxillary natural ostium and anterior ethmoidectomy by employing the visualization of 0 and angled rigid endoscope combined with various surgical instruments in all cases. If necessary, the extent of the ESS procedure could extend further to the posterior ethmoid sinus, frontal sinus, and even the sphenoid sinus according to the opacification degree of the corresponding infected sinus on computed tomography. Furthermore, the white purulent secretions were fully drained. The extensively swollen mucosa was removed as much as possible with a micro-debrider. A hemostatic sponge was placed as packing at the middle meatus in order to stop the postoperative bleeding, and the nasal packing was removed on the 7th day after surgery.

Some patients required oral surgery to remove the source of the infection and close the oral maxillary sinus communication. First, teeth with AP/PE/EPL and failed implants were extracted, followed by complete debridement of the diseased oral tissues. The method of repair was determined by the extent of bone defect of the MSF; for smaller defects, direct suture or buccal mucosal glide flap repair was performed; while for larger defects, a palatal rotational mucosal flap could be used for repair.

### 2.5. Statistical Analysis

Age and disease duration were compared as continuous data using analysis of variance. Sex, sinusitis symptoms, the extent of sinus inflammation, and the condition of bone penetration were compared using the Chi-squared test. The continuity-corrected Chi-squared test was used for theoretical frequencies T ≥ 1 and <5. Fisher’s exact probability method for theoretical frequency was utilized when T < 1. The abovementioned tests were two-sided tests with a test level of α = 0.05, i.e., *p* < 0.05, which was considered statistically significant. Statistical analyses were conducted using SPSS 23.0 (IBM Corporation, Armonk, NY, USA).

## 3. Results

### 3.1. Demographic and Clinical Characteristics of the ODS Patients

The mean age of the 103 patients was 45.87 ± 12.79 years. Sixty-three patients (61.2%) were male, and the mean duration of disease was 9.87 ± 10.57 months.

Foul nasal odor (81.6%) and nasal discharge (anterior/posterior nasal drip) (84.5%) were the two most prominent symptoms in patients with ODS. According to CT analysis, there were 80 (77.7%), 41 (39.8%), and 7 (6.8%) cases that involved the ethmoid, frontal, and sphenoid sinuses, respectively. The most common odontogenic etiologies were EPL (49.5%) and AP (32.0%) (Table 1).

There were significant differences in age (*p* = 0.002), sex (*p* = 0.036), inflammation involving the ethmoid sinus (*p* = 0.037), and bone penetration in the MSF (*p* < 0.001) among the AP, EPL, and PE groups. However, no statistically significant differences (*p* > 0.005) were found in the sinusitis symptoms among patients with different odontogenic etiologies (Table 2).

### 3.2. Review of Surgical Data

We reviewed 76 cases of simultaneous oral treatment with ESS; 71 cases were diseased tooth extractions and simultaneous wound repairs, 4 were permanent oral maxillary sinus fistula repairs, and 1 was an implant removal and wound repair. The remaining patients underwent root canal treatment (RCT) or second-stage extraction of the diseased tooth after ESS for some of the affected teeth. Among the 71 patients who underwent simultaneous extractions, 25 cases required rotating mucosal flaps on the palatal side, 21 cases required gliding flaps on the buccal side, and 25 cases required direct wound closure after filling with hemostatic sponges.

There were significant differences in preoperative and postoperative nasal symptoms and modified LKS scores. No patients developed an oral maxillary sinus fistula after the surgery. Furthermore, the sinus openings were clear, and the mucosa was not swollen at the 3-month review by nasal endoscopy (Table 3).

## 4. Discussion

ODS involves both sinusitis and odontogenic lesions; the lack of knowledge about sinusitis and oral health among dentists and otorhinolaryngologists makes the diagnosis and treatment of ODS difficult. In essence, an imbalance in the bacterial microecology leads to dysfunction in the normal clearance mechanism and drainage system of the sinuses and the symptoms of sinusitis. In a recent study, we used 16S rRNA sequencing to compare the microbiological differences between ODS patients and normal sinus secretions; the three most abundant genera were Fusobacterium, Porphyromonas, and Prevotella in ODS patients and Staphylococcus, Corynebacterium, and Cutibacterium in normal sinus secretions [20]. The presence of anaerobic bacteria in the nasal microbiome of ODS patients indicates the following: potential tissue hypoxia, or discrete microenvironment within the mucus, or bacterial biofilm in ODS patients may also be oxygen-limited, allowing anaerobic bacteria to survive. Therefore, the key to the treatment of ODS is to restore the microecological environment of the sinuses. There are two things that can be done: first, improve the drainage and anaerobic environment of the sinus cavities by medication or ESS surgery; second, completely remove the odontogenic lesions, reduce the microbial burden of the sinuses, and ultimately restore them to a stable state. Regarding the pharmacological treatment of ODS, which requires targeting specific microorganisms, a large number of studies have confirmed the following: the microbial composition of odontogenic and non-odontogenic sinusitis varies considerably; and ODS is predominantly an anaerobic infection [21,22,23,24] and is most abundant in anaerobic organisms, all of which have been associated with oral infections and have been identified in our study by high-throughput sequencing. Therefore, for ODS, we primarily use anti-anaerobic drugs, including metronidazole and Quinolones. Of course, for patients with severe microbial burdens of ODS, drug therapy alone is clearly insufficient and further surgical treatment is required. In a recently published consensus, experts agreed that a multidisciplinary approach was optimal [25]. However, concerning the optimal sequence of surgical interventions, the majority of contemporary otorhinolaryngologic literature recommends that we should primarily address the odontogenic infection source. Other authors suggest a combined approach of oral removal of dental infection and simultaneous maxillary sinus drainage via ESS [26,27,28]. ESS can be undertaken to rapidly open the sinuses and improve the patient’s symptoms, but some patients may be able to avoid surgery through dental treatment; therefore, to develop an optimal treatment plan, the etiology of odontogenic pathology should be clarified before proceeding with surgery.

**Figure 1:**
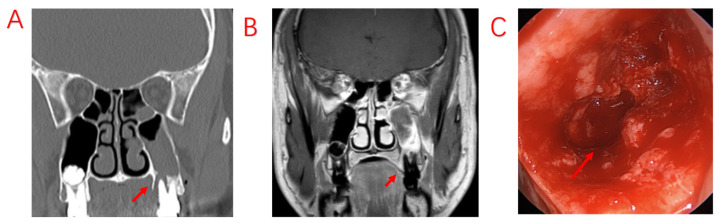
A patient with odontogenic sinusitis due to EPL with bone defects in the MSF. (**A**): Sinus CT shows resorption of alveolar bone on the palatal side of the left upper first molar, forming a narrow and deep periodontal pocket. (**B**): MRI shows diseased tissue in the periodontal pocket connected to swollen mucosa in the maxillary sinus. (**C**): Bone penetration of the palatal MSF in the extraction socket is observed during surgery. Red arrows indicate bony defects in the MSF.

Odontogenic lesions may be caused by dental disease or surgery, implant surgery, or maxillary sinus lift surgery and thus include issues whose etiologies go beyond the etymological definition of “odontogenic”. Although dental implant surgery has caused an increasing number of odontogenic diseases, the vast majority of odontogenic lesions in patients are still caused by typical dental diseases and complications of dental treatment. Among the 103 patients with ODS included in this study, the top three odontogenic etiologies were EPL (49.50%), AP (32.0%), and PE (8.70%); nearly half of the patients presented both apical and periodontal lesions, a share that differed from that reported in previous studies [29]. This discrepancy may be due to some patients having nonobvious periodontal lesions and being diagnosed with AP or no odontogenic etiology. Both pulpal and periodontal tissue lesions are involved in EPL. In patients affected by PE, EPLs usually progress slowly without important symptoms. The most common signs and symptoms of pulp-infected teeth in EPL are narrow, deep periodontal pockets that reach or approach the root apex and respond negatively to pulp vitality testing [30]. These symptoms are consistent with those in our patients diagnosed with EPL (Figure 1). According to our analysis, there are two possibilities; the first is chronic necrosis of the pulp, which occurs without considerable pain due to the chronic irritation of the pulp by the filling, resulting in apical lesions. The other possibility is pulpal necrosis due to chronic PE, which may also cause no obvious pain. Since some periapical lesions are too small to be observed on sinus CT, CBCT should be performed to look more closely at dental lesions in patients with a high clinical suspicion of ODS.

CT and CBCT have their advantages and disadvantages in the diagnosis of ODS. CT is the gold standard in the diagnosis of sinus disease due to its high resolution and ability to discern bone and soft tissue. On CT images, a full view of the sinus system can be visualized, including whether the sinonasal complex is obstructed, the extent of infection involving the sinuses, and other associated sinus diseases. However, inadequate image capture through the upper dentition and failure to thoroughly evaluate the dentition upon review of the images can lead to failure to diagnose ODS. In addition, artifacts can occur on the CT image due to fillings or metal crowns, which can interfere with the visualization of some subtle lesions [31]. Compared to CT, CBCT produces fewer streak artifacts and provides very high spatial resolution, compensating for the shortcomings of CT. Currently, CBCT serves as the standard diagnostic method for dental pathologies. Dental CBCT affords high spatial resolution and accurate detection of periapical lesions, oroantral fistulae, and periodontal diseases, especially in upper maxillary teeth [32,33]. CBCT can provide accurate parameters for digitized solutions for clinical, surgical, and restorative procedures to improve team communication and take advantage of combinations of collected data to avoid losing information using traditional manual steps [34,35]. The close relationship between the roots of the posterior maxillary teeth and the floor of the maxillary sinus is a major cause of the high prevalence of oral diseases and surgery-related mucosal disorders. The use of high-resolution CBCT in the dental profession has generated extensive research on the association of maxillary sinus inflammation with molar lesions [36,37,38,39]. In general, CT provides a comprehensive view of the changes in the soft and hard tissues of the sinuses, especially the bony changes in the wall of the floor of the maxillary sinus. CBCT focuses more on the changes in the dental tissues and details of the periodontal tissues.

**Figure 2:**
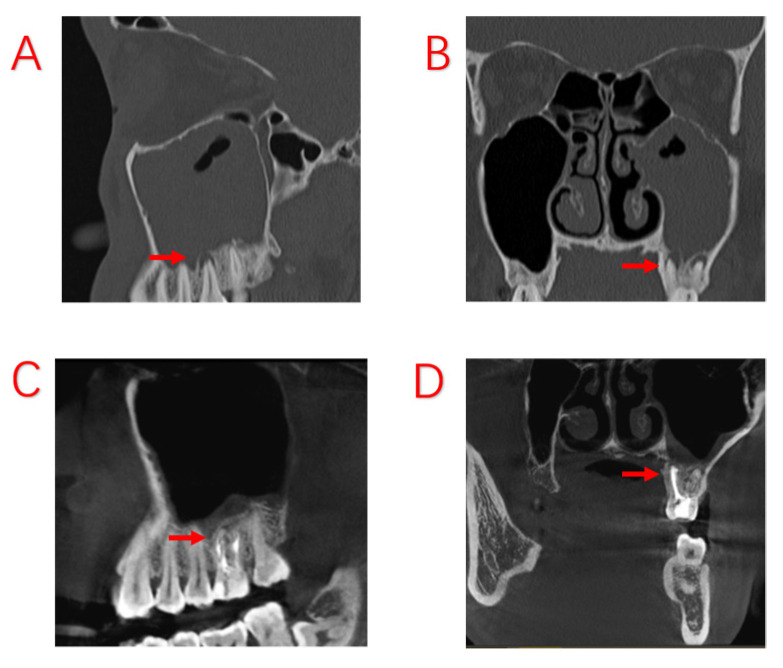
Odontogenic sinusitis due to periapical inflammation. Symptoms disappeared after root canal treatment; no ESS was performed. (**A**,**B**): Before root canal treatment, the patient developed a purulent discharge with a foul odor and obstruction of the maxillary sinus opening. (**C**,**D**): After root canal treatment, infection of the dental pulp was controlled and the symptoms of sinusitis had disappeared, with the mucosa of the maxillary sinus returning to normal. Red arrows indicate sites of apical inflammation.

**Figure 3:**
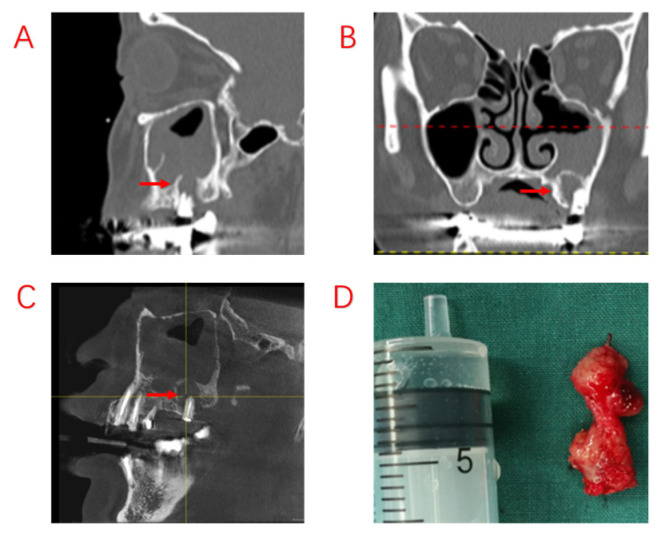
Recurrence of postoperative sinus symptoms after ESS for odontogenic sinusitis due to failure to treat the apical lesion. (**A**,**B**): Large shading of the apical area of the diseased tooth and bone penetration of the maxillary sinus floor. The maxillary sinus opening was fully opened after ESS, and the maxillary sinus mucosa could not heal completely due to the apical lesion not having been removed. (**C**): The apical lesion persisted despite RCT of the diseased tooth. (**D**): A large amount of diseased granulation tissue adhered to the apical lesion. Red arrows indicate sites of apical inflammation.

**Figure 4:**
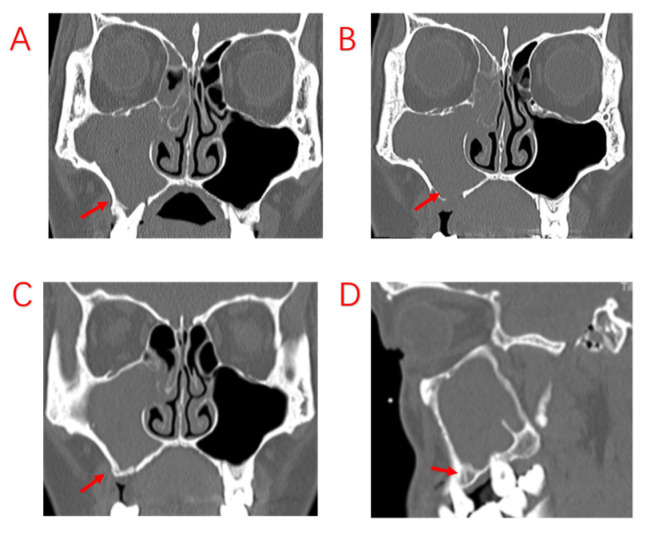
Patient undergoes tooth extraction but no sinus ESS. (**A**): The patient was found to have sinusitis of the right maxillary sinus at the time of tooth extraction, and the affected tooth was extracted without an oral maxillary sinus fistula. (**B**): The sinus CT was repeated 1 month after tooth extraction, which revealed ongoing sinus inflammation. (**C**,**D**): One year after tooth extraction, there was already new bone formation on the floor of the maxillary sinus; however, the sinus symptoms were still not relieved as the maxillary sinus was not opened. Red arrows indicate bony defects in MSF and restitution.

In this study, we compared the EPL, AP, and PE groups in terms of demographic characteristics, sinus symptoms, and extent of sinus invasion. The three groups were found to have statistically significant differences in terms of sex and age, with patients in the PE group being older. There were no statistically significant differences in sinusitis symptoms among ODS patients with different odontogenic etiologies. However, the proper treatment of odontogenic lesions can affect the overall outcome for the patient, and different treatments are required for different odontogenic causes. In a recently published consensus, experts in the survey agreed that a multidisciplinary approach was optimal [28]. We also believe that multidisciplinary collaboration is essential and have proposed different treatment approaches for different etiologies. Patients with ODS caused by AP tended to have a better prognosis, excluding those who had already had root canal treatment (RCT), root fractures, and periodontal lesions. For those patients whose lesions were limited to the apices of the teeth, infection from the pulp could be blocked by RCT, potentially avoiding the ESS procedure. Therefore, it is recommended that patients with ODS caused by periapical lesions undergo dental treatment first. The infection from the pulp was controlled with RCT, and the patients’ sinus symptoms subsequently improved as early as during the RCT procedure (Figure 2). After RCT, the decision to perform ESS was based on sinusitis symptoms and radiological findings. If the lesions are more extensive, involving root bifurcation, root fracture, or periodontal inflammation, extraction of the diseased tooth should be considered to completely remove the lesion (Figure 3). In some of our cases, the diseased teeth were extracted before the ESS procedure, but the sinusitis symptoms did not improve even after the mucosa in the oral cavity had healed. Although the pathological irritation from the oral cavity was resolved, the obstruction of the sinus openings resulted in the formation of an anaerobic environment in the maxillary sinuses, requiring the ESS procedure to improve sinus ventilation and drainage (Figure 4).

**Figure 5:**
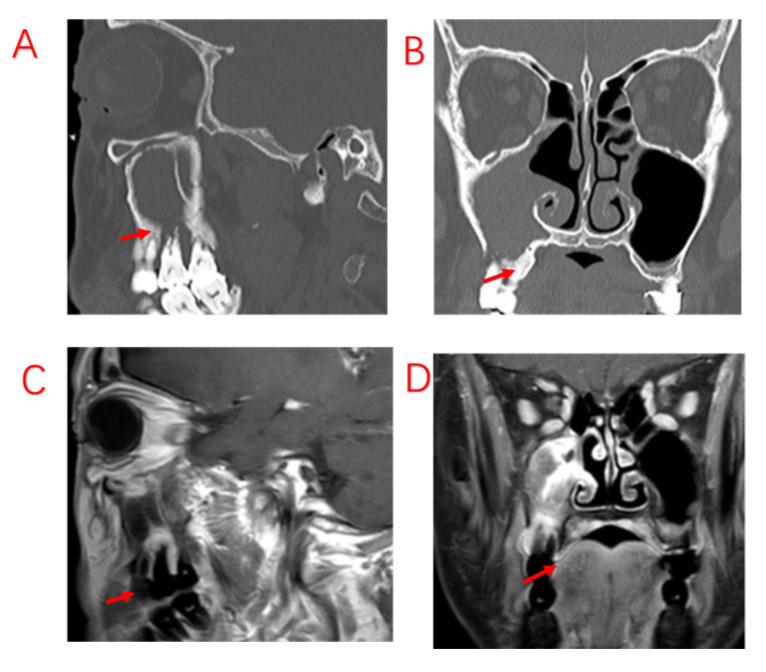
Patient with severe periodontal infection and no improvement in sinusitis symptoms after ESS. (**A**,**B**): Sinus CT shows adequate maxillary sinus opening, alveolar bone resorption of the diseased tooth, and bone defects in the MSF. (**C**,**D**): MRI shows periodontal lesion tissue attached to the mucosa of the maxillary sinus, with significant swelling of the maxillary sinus mucosa. Red arrows indicate specific sites of bony defects in MSF.

For patients with severe periodontal infection, it is difficult to improve the resorbed alveolar bone through treatment. Infection from the oral cavity will continue to irritate the maxillary sinus mucosa through the resorbed and destroyed alveolar bone. A total of 5 of the 103 patients had undergone ESS surgery, and although the sinus orifices were adequately opened, the inflammation of the sinus mucosa did not improve after surgery because the cause of the oral cavity was not addressed during the first treatment (Figure 5). During the second operation, we found no obvious purulent secretions in the maxillary sinus, but considerable mucosal swelling, which was associated with persistent irritation from the oral lesion, was found. Therefore, the oral lesion was removed during the surgery, and the symptoms completely disappeared. Hence, it is concluded that the best treatment plan for patients with EPL and PE with severe periodontal pathology, especially those with bone penetration in the MSF, should be sinus surgery combined with extraction of the affected teeth, removing the oral lesion, opening the maxillary sinus for drainage, and closing the oral maxillary sinus fistula as needed. In the authors’ opinion, there are two criteria for assessing the impact of odontogenic lesions in ODS surgical treatment. The first is whether the bone of the MSF has been penetrated. The second is whether the diseased tooth shows severe periodontal bone destruction and resorption. If both of these two criteria are met, ESS combined with the removal of the dental lesions is the best choice.

However, there are some limitations in the current study. First, the sample size was relatively small. Second, this was a single-center study in China, where the distribution of the odontogenic etiologies of ODS might differ from that in Europe and the United States, as such distribution is related to the local level of oral health knowledge. Further studies investigating these aspects will thus be needed in the future.

## 5. Conclusions

The key to the treatment of ODS is restoration of the microecological environment of the sinuses. Accurate evaluation and diagnosis of oral lesions are essential to the development of a treatment plan. Patients who develop symptoms of sinusitis have severe sinus mucosal lesions and develop sinus orifice obstruction with purulent discharge as a result of mucosal inflammation and, to a certain degree, epithelial dysfunction. If an early oral therapeutic strategy can be taken to restore the sinus’s microecological environment, some patients can avoid undergoing ESS surgery. EPL is a neglected oral etiology with a destructive effect on the bone of the MSF, and sinus surgery should be performed concurrently with extraction of the diseased teeth and debridement of the lesion.

## Figures and Tables

**Table 1:** Demographics and clinical variables in patients with ODS.

Variables	Symptomatic Patients with ODS (*n* = 103)
Age (mean ± SD, years)	45.87 ± 12.79
Male sex (*n*, %)	63 (61.2%)
Disease duration (mean ± SD, months)	9.87 ± 10.57
Symptoms (*n*, %)	
Foul nasal odor	84 (81.6%)
Nasal obstruction	60 (58.3%)
Nasal discharge (anterior/posterior nasal drip)	87 (84.5%)
Facial pain/pressure	63 (61.2%)
Reduction or loss of smell	14 (13.6%)
Sinuses involved (*n*, %)	
Maxillary sinus	103 (100.0%)
Ethmoid sinus	80 (77.7%)
Frontal sinus	41 (39.8%)
Sphenoid sinus	7 (6.8%)
Odontogenic causes (*n*, %)	
AP	33 (32.0%)
EPL	51 (49.5%)
PE	9 (8.7%)
Oroantral communications and fistulas (temporary)	5 (4.8%)
Oroantral communications and fistulas (persistent)	4 (3.9%)
Postoperative dental implants	1 (1.0%)

SD, standard deviation; AP, apical periodontitis; EPL, endo-periodontal lesions; PE, periodontitis.

**Table 2:** Comparison of the characteristics between the apical periodontitis (AP), endo-periodontal lesion (EPL), and periodontitis (PE) groups.

Variables	AP (*n* = 33)	EPL (*n* = 51)	PE (*n* = 9)	*p* Value
Age (mean ± SD, years)	40.00 ± 12.16	48.92 ± 12.08	50.78 ± 6.82	0.002 ^a^
Male sex (*n*, %)	14 (42.4%)	36 (70.6%)	5 (55.6%)	0.036 ^b^
Course of disease (mean ± SD, months)	10.42 ± 11.35	10.23 ± 11.14	11.11 ± 9.21	0.975 ^a^
Symptoms (*n*, %)				
Foul nasal odor	27 (81.8%)	40 (78.4%)	9 (100.0%)	0.304 ^b^
Nasal obstruction	20 (60.6%)	30 (58.8%)	6 (66.7%)	0.905 ^b^
Nasal drip	28 (84.8%)	46 (90.2%)	7 (77.8%)	0.427 ^c^
Facial pain/pressure	21 (63.6%)	30 (58.8%)	7 (77.8%)	0.547 ^b^
Reduction or loss of smell	5 (15.2%)	8 (15.7%)	1 (11.1%)	1.000 ^c^
Involving sinuses (*n*, %)				
Maxillary sinus	33 (100.0%)	51 (100.0%)	9 (100.0%)	-
Ethmoid sinus	30 (90.9%)	37 (72.5%)	5 (55.6%)	0.037 ^b^
Frontal sinus	17 (51.5%)	19 (37.3%)	1 (11.1%)	0.077 ^b^
Sphenoid sinus	2 (6.1%)	3 (5.9%)	1 (11.1%)	0.678 ^c^
Bone penetration of MSF	15 (45.5%)	44 (86.3%)	5 (55.6%)	<0.001 ^b^

^a^ ANOVA; ^b^ χ^2^ test; ^c^ Fisher’s exact test; SD, standard deviation; MSF, maxillary sinus floor.

**Table 3:** Patients’ pre-operation and post-operation sinusitis symptoms and nasal endoscopic findings.

Symptoms and Nasal Endoscope Finding (*n* = 103)	Pre-Operation	Post-Operation	*p* Value
Symptoms			
Foul nasal odor	84 (81.6%)	0 (0%)	<0.001
Nasal obstruction	60 (58.3%)	3 (2.9%)	<0.001
Nasal discharge	87 (84.5%)	6 (5.8%)	<0.001
Facial pain/pressure	63 (61.2%)	0 (0%)	<0.001
Nasal endoscopy finding			
Edema			
0: absent	11 (10.7%)	86 (83.5%)	<0.001
1: mild	43 (41.7%)	12 (11.6%)	<0.001
2: severe	49 (47.6%)	5 (4.9%)	<0.001
Polyps			
0: no polyps	69 (67.0%)	103 (100%)	<0.001
1: polyps in middle meatus only	19 (18.4%)	0 (0%)	<0.001
2: beyond middle meatus	15 (14.6%)	0 (0%)	<0.001
Discharge			
0: no discharge	9 (8.7%)	92 (89.3%)	<0.001
1: clear, thin discharge	5 (4.9%)	8 (7.8%)	0.39
2: thick, purulent discharge	89 (86.4%)	3 (2.9%)	<0.001

## Data Availability

Available on request.
